# Susceptibility status of major malaria vectors to novaluron, an insect growth regulator South-Eastern Tanzania

**DOI:** 10.11604/pamj.2022.41.273.33793

**Published:** 2022-04-05

**Authors:** Amos Justinian Ngonzi, Letus Laurian Muyaga, Halfan Ngowo, Naomi Urio, John-Mary Vianney, Dickson Wilson Lwetoijera

**Affiliations:** 1 Ifakara Health Institute, Environmental Health and Ecological Science Department, PO Box 53, Ifakara, Tanzania,; 2 The Nelson Mandela African Institution of Science and Technology, Department of Life Science and Bio-Engineering, PO BOX 447, Arusha, Tanzania

**Keywords:** Insect growth regulator, novaluron, susceptibility, An. arabiensis, An. gambiae, An. funestus, Tanzania

## Abstract

**Introduction:**

application of Insect Growth Regulator (IGR) such as pyriproxyfen has shown a promising result in controlling malaria transmitting mosquitoes through autodissemination technique. Novaluron that inhibits the chitin development at mosquito larval stage present a promising candidate IGR for rotation with pyriproxyfen to prevent a chance of resistance development. This study assessed the susceptibility of immature stages of Anopheles arabiensis, Anopheles gambiae and Anopheles funestus to novaluron.

**Methods:**

susceptibility bioassays using technical grade novaluron (98% active ingredient) were performed inside the semi-field system using first instar larvae of Anopheles species. For each tested species, a total of 1500 larvae were used in the bioassay. Concentration range of 0.01 mg/l to 2 mg/l of novaluron were tested to establish Lethal Concentration (LC) sufficient to kills 50%, 90% and 99% of the exposed larvae by using log-dose response analysis.

**Results:**

of the tested mosquitoes, Anopheles gambiae were highly susceptible to novaluron followed by An. Arabiensis and then An. funestus. Lethal concentrations, LC_50_, LC_90_ and LC_99_ (95%CI) in mg/l for An. gambiae were 0.018, 0.332 and 2.001 respectively. For An. arabiensis were 0.026, 0.546 and 2.013; and for An. funestus were 0.032, 1.00 and 5.580. High larval mortality was recorded at high concentration (2mg/L), with 80% mortality within 3 days post exposure.

**Conclusion:**

the study demonstrates the efficacy of novaluron in controlling Anopheles mosquito species at immature stages via larval mortality. These findings warrant further testing of novaluron for autodissemination by different vector species for its inclusion in rotation to prevent development of resistance.

## Introduction

Outdoor and indoor malaria transmissions have profoundly led to the present malaria morbidity and mortality. In the year 2020 alone, there was 241 million malaria cases and 627,000 deaths globally [1]. Disproportionately, countries in sub-Saharan Africa, including Tanzania, have continue to accounting 95% of total cases and 602,000 deaths [1]. Additionally, malaria is considered to be a major economic burden in Africa, whereby the continent lost 12 billion USD in year 2000 [2]. It was demonstrated that, 10% global decrease in malaria incidence can result up to 0.3% average increase in income per capita, with high malaria endemic areas benefiting most [3,4]. With this economic impact, malaria prevention strategies are highly needed for implementation to overcome the burden.

Different strategies for malaria prevention with vector control tools, primarily the long-lasting insecticide mosquito nets (LLINs) and indoor residual sprays (IRS) are strongly recommended by the Word Health Organization [1]. Across sub-Saharan Africa where >90% of the disease burden is concentrated, both LLINs and IRS have significantly suppressed malaria vectors, especially, those that bite and rest indoors. These preventions and controls measures have contributed nearly 40% of 57% reduction of clinical disease incidences [5]. However, rapid increase in insecticide resistance and observed higher outdoor biting and resting patterns of malaria vectors jeopardize future application of these interventions towards malaria elimination efforts [6]. These challenges demonstrate urgent need for alternative malaria vector control measures which can complement the existing malaria vector interventions.

Larval source management, is another vector control tool that uses chemical and biological agents used to control malaria vectors in aquatic stages [7,8]. This technique works by reducing vector densities at mosquito breeding habitats either through killing effect of mosquito immature stages or adult emergence inhibition effect [7,8]. Despite of the success attained by larviciding for controlling malaria vectors across Africa, high operational cost and low coverage of the targeted breeding habitats remain to be the greatest challenges [9-12].

Of importance, mosquito assisted larviciding commonly known as autodissemination with insect growth regulators (IGRs) (i.e pyriproxyfen and novaluron) can accurately target aquatic habitats with larvicide and deliver the desired impact [13-16]. By definition, auto-dissemination is the management method in which insects such as mosquitoes get exposed and pick biological or chemical insecticide such as IGRs while seeking the host or feeding or resting, and transfer lethal concentrations vertically or horizontally to the oviposition sites and result in reduction of adult mosquitoes [13,17].

Novaluron is an IGR that have been recently tested using autodissemination technique against different mosquito species [16]. It inhibits the chitin synthesis process at larval stages of mosquitoes through contact and ingestion of a benzoylphenyl urea formation, whereby larvae succumb to death as the results of abnormal endocuticle deposition [16,18-20]. Of interest, novaluron has a reduced risk to the environment including mammals, birds, aquatic animals and non-targeted insects [19,20].

Recently, novaluron has shown effect in reducing adult mosquito density at their larval stage. With evidences reported on its efficacy, novaluron has excellently worked against immature *Anopheles quadrimaculatus, Aedes aegypti* and *Culex quinquefasciatus* in the laboratory and field settings [16,21,22]. Likewise, using autodissemination technique, Swale *et al*. 2018 demonstrated the effect of novaluron against *An. quadrimaculatus*, causing up to 22% reduction in adult emergence as the results of larval mortality [16]. Despite the benefits that novaluron offers to control other mosquito borne diseases, there is no evidence of its application to control the main malaria vectors in rural South-eastern Tanzania. Here, we evaluated the susceptibility of the main malaria vectors in South-eastern Tanzania, *Anopheles arabiensis, Anopheles gambiae* and *Anopheles funestus*, to varying doses of novaluron under semi-field setting.

## Methods

**Figure 1:**
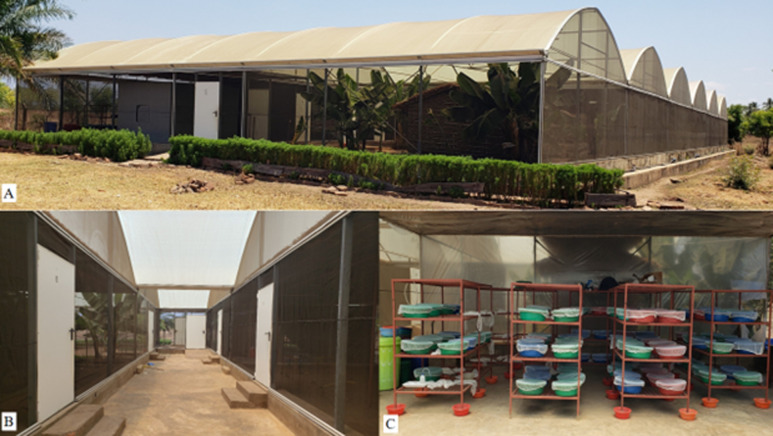
A) semi-field system used in experiments; B) chambers inside the semi-field; C) mosquito rearing insectary inside semi-field system

**Study site:** this study was conducted in a Semi-Field System (SFS) of Ifakara Health Institute between July-September, 2021. The experimental SFS is located at Kining´ina village (8.11417°S, 36.67484°E) in Ifakara, Kilombero District, South-eastern Tanzania (Figure 1). Detailed description and dimensions of the SFS has been described elsewhere [14,23].

**Mosquitoes:** the study used insectary reared mosquitoes from the established colonies of *An. arabiensis, An. gambiae* and *An. funestus*. Details of colonies´ rearing and maintenance procedures are provided elsewhere [23-26]. All bioassays used first instar larvae owing to its high susceptibility to novaluron [16].

**Preparation of test concentrations:** test concentrations of novaluron, 98% test concentration (technical materials; Jiaozuo Huisell Chem, Ltd, China) were prepared using standardized procedures [27]. Mass of novaluron; 0.01mg, 0.05mg, 0.1mg and 2.0 mg were measured using electronic beam balance and dissolved in 1000 ml of tap water to prepare the concentrations; 0.01mg/L, 0.05mg/L, 0.1mg/L and 2mg/L respectively. Aliquots of 200 ml of each prepared concentration was placed in plastic cup (four replicates) for bioassays plus four control plastic cups containing tap water alone.

**Laboratory susceptibility test:** the bioassays had control and treatment cups containing test concentration and mosquito larvae. The expected outcome was larval mortality at the treatment cups compared to the control cups to confirm lethal concentrations that is required to kill 50%, 90% and 99% of exposed larvae. Twenty-five (25) first instar larvae per replicate were exposed to novaluron concentrations; 0.01mg/L, 0.05mg/L, 0.1mg/L and 2.0 mg/L. The set-up was repeated three times on different days to counter confounders in the bioassay. Larvae were fed at 1-day interval with Tetramin® fish food throughout the course of the assay. The larval mortality was monitored on 24 hours interval until all larvae were dead or pupated. Dead larvae were counted and removed from the plastic cups. The larval mortality data was corrected using Abbott´s formula. Log-dose response analysis was carried out to determine lethal concentration of 50%, 90% and 99% (LC_50_, LC_90_ and LC_99_). The temperature during the assay ranged between 24-27°C, 80% ± 10% relative humidity and the photoperiod of 12L: 12D. Diagnostic concentration was established from the lethal concentrations that killed up 99% of the exposed *Anopheles* larvae, and it was defined as the two times of LC_99_ [27].

**Effect of novaluron on pupation rate:** the effect of novaluron on larval mortality was recorded to determine the percentage inhibition of pupation (PI%). Moribund and dead larvae and pupae that did not completely separated from the larvae case, were considered as affected by novaluron. The experiment ended 15 days post-exposure. The data from all replicates were combined to calculate the mean of affected larvae. The PI% of *Anopheles* larvae caused by novaluron was calculated using the formula:


$$PI\%=100-\left(\frac{T\times 100}{C}\right)$$

Whereby; T= percentage pupation in treated cups; C= percentage pupation in control cups.

**Statistical analysis:** data were analyzed using R software (Rv-4.1.1) [28]. Generalized linear mixed models were used to assess the proportion of dead larvae for each concentration [29]. The proportion of dead larvae were modelled as a response variable and test concentrations were considered as fixed effect while replicates and days were included as a random term to account for the pseudo replicates and unexplained variation between days. Lethal concentrations, LC_50_, LC_90_ and LC_99_ were determined using log-dose response analysis from *dose-response curve* package [30]. The curve was used to determine the desired concentration of novaluron. Additionally, Tukey honest significance test (TukeyHSD) was used to assess the pairwise difference between different concentration levels. Risk ratio and their corresponding 95% CI were reported, whereby, the statistical significance was considered when p-values ≤ 0.05.

**Ethical consideration:** prior to laboratory work the research proposal was presented to the Nelson Mandela Institute of Science and Technology and Ifakara Health Institute for approval. Further, the ethical approval for the study was granted by Institutional Review Board of Ifakara Health Institute (IHI/IRB/No: 20-2021).

## Results

**Figure 2:**
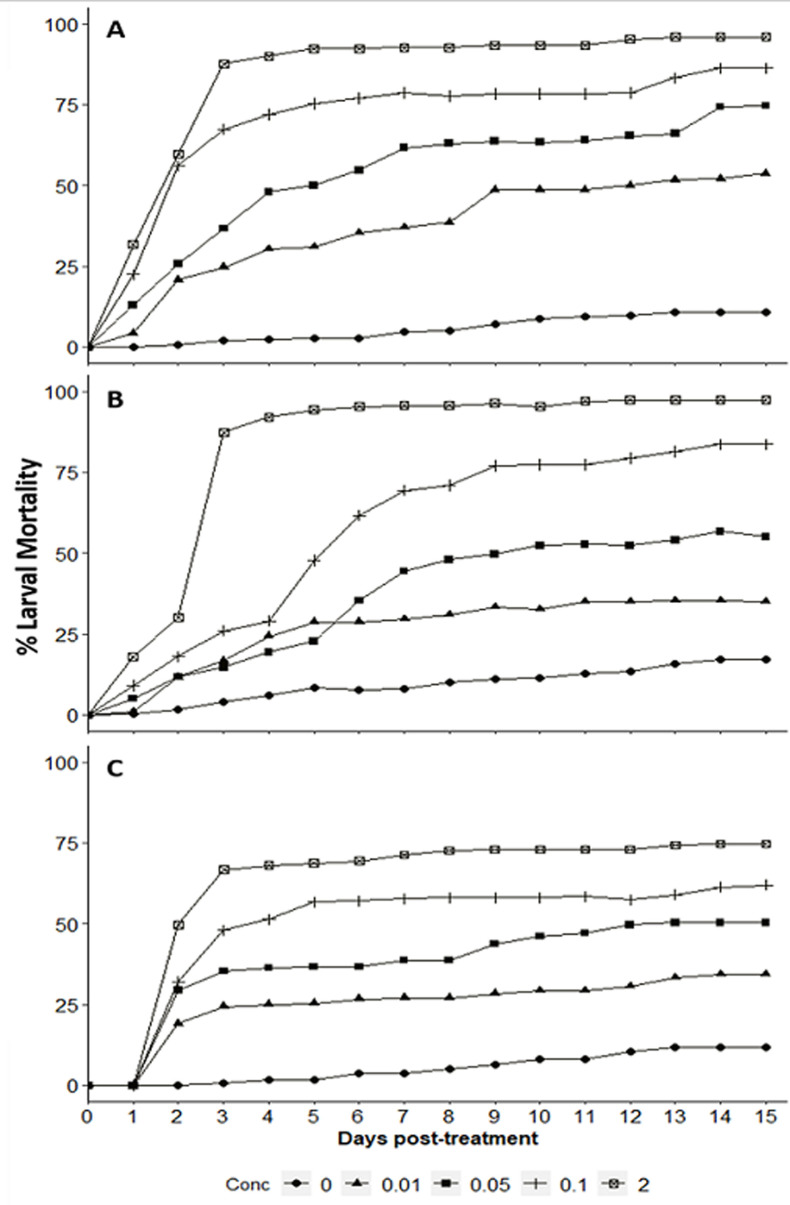
cumulative mortality percentage of: A) *An. gambiae*; B) *An. arabiensis* and C) *An. funestus* larvae when 1^st^ instar larvae were treated with novaluron-chitin synthetic inhibitor

**Figure 3:**
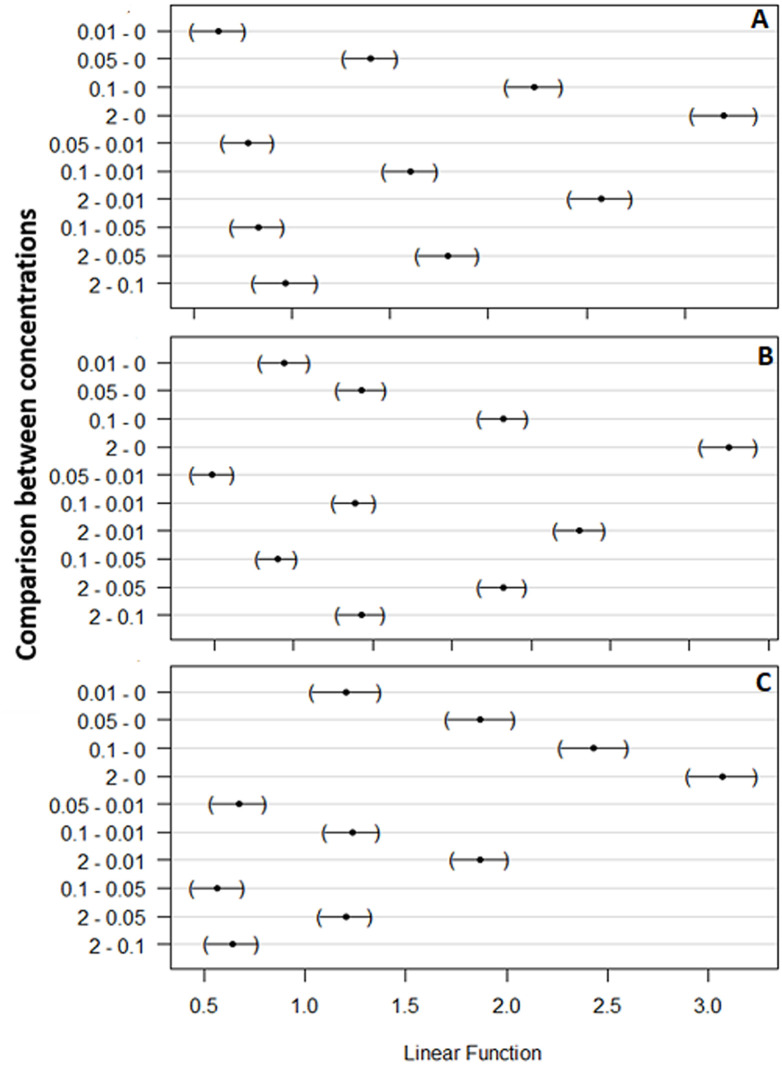
results of pair-wise post hoc comparison using Tukey's honestly significance tests (Tukey's HSD); similarities and differences between larvae mortality at different concentrations; A) *An. gambiae*; B) *An. arabiensis* and C) *An. funestus*

**Laboratory susceptibility test:** high larval mortality was recorded with high concentrations of novaluron, whereas low concentrations were associated with delayed mortality. *An. gambiae* larvae were more susceptible with LC_50_ and LC_90_ being 0.0179 mg/L and 0.332 mg/L respectively, while LC_50_ and LC_90_ for *An. arabiensis* and *An. funestus* was 0.02561 mg/L and 0.5460 mg/L; and 0.0323 mg/L and 1.000mg/L respectively (Table 1). Larval mortality in the respective control ranged from 7% to 15% depending on mosquito larvae species exposed (Figure 2). However, the laboratory susceptibility test yielded the diagnostic concentrations for all three-target species (Table 1). The results on the comparison of different concentrations on the larval mortality are summarized in Table 2. In three *Anopheles* species, *An. gambiae* was highly susceptible [RR = 1.0842, p < 0.005], followed by *An. arabiensis* [RR = 10.237, p < 0.001], and *An. funestus* [RR = 11.41, p < 0.001] at test concentration of 0.1 mg/L (Table 2). However, the pair-wise comparison test using Tukey´s HSD showed significant difference between control and 0.1 mg/L for *An. arabiensis* (z = 42.83, p < 0.001), *An. gambiae* (z = 43.87, p < 0.001) and *An. funestus* (z = 40.53, p < 0.001) (Figure 3).

**Table 1:** larval susceptibility of malaria vector species to novaluron

Species	LC_50_ (mg/L)	95%CI	LC_90_ (mg/L)	95%CI	LC_99_ (mg/L)	95%CI	Diagnostic Conc. (mg/L)
*An. gambiae*	0.018	0.016,0.020	0.332	0.168,0.496	2.001	1.986,3.206	4.002
*An. arabiensis*	0.026	0.027,0.038	0.546	0.374,0.719	2.013	1.997,4.491	4.026
*An. funestus*	0.032	0.021,0.03	1.000	0.467,1.535	5.580	4.687,8.496	11.160

**Table 2:** larvae mortality and their risk effects at different concentrations of novaluron

Species	Conc. (mg/L)	Predicted mean (95%CI)	RR (95% CI)	P-value
***An. gambiae***	0.00	0.318 (0.149,0.675)	1	
	0.01	0.595 (0.280,1.265)	0.518 (0.384,1.349)	0.177
	0.05	1.293 (0.609,2.745)	0.257 (0.384,0.668)	0.504
	0.10	2.957 (1.391,6.284)	1.084 (0.385,2.819)	0.004
	2.00	7.786 (3.656,16.582)	2.052 (0.386,5.321)	< 0.001
***An. arabiensis***	0.00	0.144 (0.116,0.178)	1	
	0.01	0.369 (0.299,0.454)	2.567 (2.300,2.865)	< 0.001
	0.05	0.600 (0.488,0.737)	4.174 (3.751,4.644)	< 0.001
	0.10	1.471 (1.197,1.808)	10.237 (9.204,11.357)	< 0.001
	2.00	6.121 (4.939,7.588)	42.604 (37.718,48.122)	< 0.001
***An. funestus***	0.00	0.096 (0.044,0.211)	1	
	0.01	0.319 (0.145,0.699)	3.325 (2.947,3.752)	0.004
	0.05	0.622 (0.284,1.362)	6.487 (5.767,7.298)	< 0.001
	0.10	1.094 (0.500,2.396)	11.41 (10.145,12.839)	< 0.001
	2.00	2.067 (0.944,4.528)	21.56 (19.119,24.306)	0.070

CI = confidence interval, RR = risk ratio. Control used as reference RR = 1, the predicted means were derived from generalized linear model which is the average of larvae dead in each concentration

**Figure 4:**
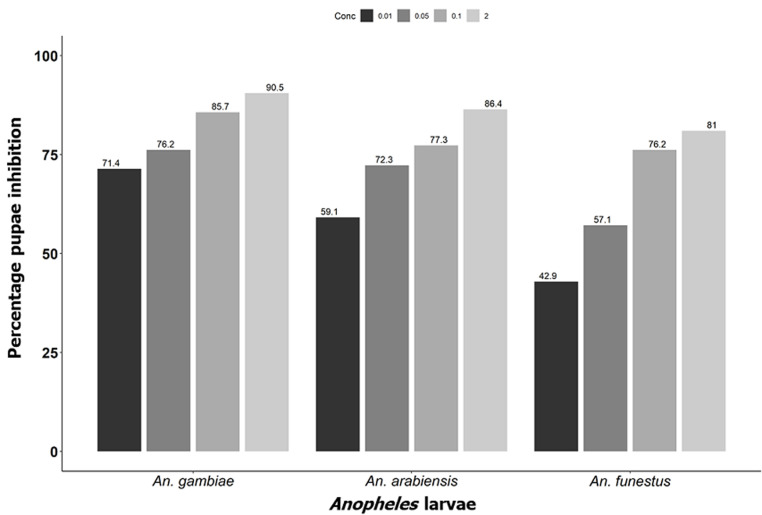
percentage inhibition of pupation of different malaria vectors at different test concentration of novaluron 15 days post-treatment

**Effect of novaluron on pupation rate:** the results demonstrated high percentage inhibition of pupation with increase in concentration. Highest PI% was recorded at 2 mg/L compared to other low concentrations across all three *Anopheles* species with PI% of 90.5%, 86.4% and 81.0% for *An. gambiae, An. arabiensis* and *An. funestus* respectively (Figure 4).

## Discussion

This study demonstrated up to 80% and 90% larval mortality and pupae inhibition of the exposed larvae of *Anopheles gambiae, Anopheles arabiensis* and *Anopheles funestus*, an afro-tropical malaria vectors to novaluron under controlled settings. These findings corroborate other previous reports that demonstrated the control of *Anopheline, Adenine* and *Culicine* mosquitoes using novaluron under laboratory and field settings [16,18,31]. Lethal concentrations sufficient to kills 50% and 90% of the exposed mosquito larvae were different across three tested species; all achieved within 15 days post-exposure. This highlights delayed developmental duration of exposed larvae as the results of novaluron effect [18,32,33]. Over 50% mortality of all *Anopheles* larvae were observed in between 2^nd^ and 3^rd^ day post-exposure at maximum test concentration of novaluron (2mg/L). Despite of the development of the exposed larvae to 3^rd^ instar, none was able to reach 4^th^ instar or pupae stage. Previous studies assessing the effect of novaluron to mosquito larvae have also reported slow and extended larval growth and delayed mortality post-exposure time [16,18,21]. This delayed mortality is expected to reduce pressures on mosquitoes to develop resistance to the novaluron, and offer a more sustainable insecticide for vector control thereof [21,32].

In comparison, *An. gambiae* was more susceptible to novaluron followed by *An. arabiensis* and lastly *An. funestus*. Lethal concentrations of novaluron required to kill 50%, 90% and 99% of *An. funestus* larvae was one to two and half times higher than that for *An. gambiae*and *An. arabiensis*. In addition, the diagnostic concentration for *An. funestus* (11.160 mg/L) was three times higher than that of *An. gambiae* (4.002 mg/L) and *An. arabiensis* (4.026 mg/L). Although not investigated under this study, the probable cause for reduced susceptibility might be a high level of pyrethroids resistance in *An. funestus* documented by other studies in the same study location [34,35]. A single study, has also highlighted possibility of cross-resistance between pyrethroids and insect-growth regulators within *Anopheles* population, which might be applicable in this case [36]. The difference of lethal and diagnostics concentrations recorded under different studies might be explained by physiological difference with test species [16,18].

There is increasing evidence that the use of IGRs of different mode of action against mosquitoes can counteract and/or delay the development of insecticide resistance [8,37]. These findings point out the efficacy of novaluron in reducing adult mosquito population at breeding habitats. Thus, an additional insecticide that may be applied in rotation with other IGRs, such as pyriproxyfen to manage insecticide resistance and reduce adult mosquito population at their larval habitats. Of importance, World Health Organization (WHO) approval on the use of novaluron in drinking water signals its safety to human and animals, and warrant its testing using conventional larviciding or autodissemination techniques in different settings [38].

This study had a number of limitations; under laboratory settings no attempt was made to test for persistence of novaluron in the test cups beyond single larval exposure. While low susceptibility of *An. funestus* to novaluron was attributed to its high insecticide resistance status, no actual experiments that were carried to ascertain this assertion, and this represent another study limitation. Therefore, these limitations add on the list of future studies towards development of novaluron as the potential larvicide for malaria vector control.

## Conclusion

This study conclude that major malaria vectors found in Kilombero, Tanzania are susceptible to novaluron at low concentration. This is the first demonstration on the susceptibility of *An. gambiae, An. arabiensis, An. funestus* to novaluron under laboratory settings. These results warrant further semi-and field testing of novaluron using the autodissemination technique against *An. gambiae, An. arabiensis* and *An. funestus* for its inclusion in rotation to prevent evolution of insecticide resistance.

### What is known about this topic

- Susceptibility of other mosquito species of disease importance to novaluron;
- Possibility of autodissemination of novaluron by other mosquitoes such as Anopheles quadrimaculatus.

### What this study adds

- Demonstrated that major malaria vectors in Tanzania are highly susceptible to low dosages of novaluron;
- Offer empirical evidence on novaluron as an additional IGR for malaria vector control at its aquatic habitats, that can be considered for application in rotation with other IGR such as pyriproxyfen (PPF), to manage insecticide resistance development.
